# Active *Helicobacter pylori* Infection Does Not Influence Outcomes After Sleeve Gastrectomy—Observational Cohort Study

**DOI:** 10.3390/jcm14010109

**Published:** 2024-12-28

**Authors:** Martyna Łukasiewicz, Patryk Lisovski, Monika Proczko-Stepaniak, Maciej Wilczyński, Marzena Szarafińska, Dariusz Świetlik, Michał Szymański

**Affiliations:** 1 Department of Oncological, Transplant and General Surgery, Faculty of Medicine, Medical University of Gdansk, 80-214 Gdansk, Poland; martyna.lukasiewicz@gumed.edu.pl (M.Ł.);; 2 Department of Emergency Medicine, Faculty of Health Sciences with the Institute of Maritime and Tropical Medicine, Medical University of Gdansk, 80-214 Gdansk, Poland; 3 Division of Biostatistics and Neural Networks, Medical University of Gdansk, 80-211 Gdansk, Poland

**Keywords:** bariatric surgery, helicobacter pylori, complications, sleeve gastrectomy, eradication

## Abstract

**Background**: *Helicobacter pylori* (HP) is under investigation for its potential role in postoperative complications. While some studies indicate no impact, they often cite short or incomplete follow-up. This study aims to compare 1-year outcomes in groups with and without active HP infection after bariatric surgery, also assessing HP prevalence in postoperative specimens of sleeve gastrectomy (SG) patients. **Methods**: Conducted between May 2020 and May 2021, this study involves both retrospective and prospective data collection from 93 eligible SG patients. Demographics, surgical outcomes and follow-up results (including complications; weight and BMI at 3, 6 and 12 months post-SG) were extracted. **Results**: No statistically significant differences in body weight were observed at 3 (*p* = 0.3757), 6 (*p* = 0.1422) or 12 (*p* = 0.2737) months post-surgery between the HP-positive (Group A) and non-infected (Group B) groups. Group A experienced significant reductions in body weight at 6 and 12 months (*p* < 0.0001), while group B showed significant reductions at 3, 6 and 12 months (*p* < 0.0001), with additional decreases at 6 and 12 months post-surgery compared to 3 months. No significant differences in overall surgery-related side effects were observed between the groups. **Conclusions**: Patients with active HP infections undergoing SG displayed comparable rates of short- and long-term complications to the non-infected group. Active HP infection did not impact body weight loss outcomes at 12 months, but it may potentially slow it down in the initial three-month post-surgery period. This underscores the need to consider eradication while maintaining awareness of the potential side effects associated with the process.

## 1. Introduction

Obesity and overweight are defined by the WHO as an abnormal or excessive fat accumulation that presents a risk to health. It is further specified that an overweight person has a body mass index (BMI) greater than or equal to 25 kg/m^2^, and a person with obesity greater than or equal to 30 kg/m^2^ [1].

Both obesity and overweight are primarily characterized by an excess accumulation of fat in the body. However, the risk to health is largely attributed to the metabolic alterations induced by the adipokines secreted by the excess fat tissue. These metabolic changes can lead to various health conditions, including hypertension, diabetes and obstructive sleep apnea [2,3].

Both conditions also currently constitute rapidly growing problems worldwide, with 1.9 billion adult people being overweight in 2016, according to the WHO; of that number, 650 million were obese [1]. A comparison of the local data in Poland, compiled by the Main Statistical Office, shows that the obesity rate in the Polish population increased from 16.7% to 18.5% between 2014 and 2019 [4].

The most common strategy for the management of obesity is diet control, with the support of pharmacological interventions at the initial stage. If those methods prove to be inadequate, in certain cases, bariatric surgery (BS) is known to be the most effective option [5,6]. Currently, the most common surgery is laparoscopic sleeve gastrectomy (SG), based on both effectiveness and predictable outcomes [5,7].

Even though there are well-known risk factors, such as smoking, which are proven to increase the complication rates of BS, some factors are of unknown significance.

One of these is gastritis caused by the bacterium *Helicobacter pylori* (HP), which might potentially lead to postoperative complications, changes in glycemia after surgery or peptic ulcer disease [5,8,9,10,11]. HP is a Gram-negative bacterium, known for causing gastric mucus inflammation and for being an etiological factor in mucosa-associated lymphoid tissue (MALT), cancers and peptic ulcer disease [8,12]. Moreover, recent studies suggest that H. pylori can also influence extra-gastric aspects, including lower gastrointestinal symptoms and immunological tolerance phenomena induced by the bacterium, highlighting its broader systemic impact [13]. According to population studies, the prevalence of the infection is often higher than 50% within Southern and Eastern Europe, South America and Asia [12]. Regarding the above-listed conditions, the eradication of HP is implemented at an asymptomatic stage of the infection [8].

All patients undergoing bariatric surgery are expected to first go through preoperative preparation consisting of several specialist consultations and procedures [14]. In recent years, preoperative endoscopic ultrasonography (EUS) has gained significance for assessing potential gastric pathologies [15]. Among the standard procedures is gastroscopy, during which a biopsy of gastric mucosa is performed [16,17]. The specimen is used for histopathologic examination and tested for HP [17]. Patients with a positive HP test result must undergo an eradication protocol based mostly on a protocol of three antibiotics and proton pump inhibitors [18]. Nevertheless, sometimes the eradication is ineffective due to antibiotic resistance, advanced age, smoking, insulin resistance, diabetes or an unsuitable dosage for the patient’s body weight [19,20].

However, some studies have reported that HP has no impact on postoperative complications following BS while underscoring the fact of short or incomplete follow-up [21,22].

There is limited information regarding the topic of the influence of confirmed active intraoperative HP infection on body weight loss following BS and the long-term complication rate. Therefore, the primary aim of this study was to compare the 1-year follow-up outcomes between a group operated on while having active HP infection and a group without infection. The secondary outcome was to assess the prevalence of HP infection in postoperative specimens in a group of bariatric patients who underwent SG.

## 2. Materials and Methods

### 2.1. Subjects

Between May 2020 and May 2021, a total number of 265 bariatric procedures were performed at the bariatric center, among which 144 were SG procedures. A total of 51 patients were excluded from further analysis, mostly due to incomplete follow-up. Therefore, a total of 93 patients were enrolled in the study. During the study period, one nationwide lockdown was instituted between 26 March 2021 and 5 May 2021 (40 days) due to the COVID-19 pandemic. All patients were qualified in accordance with the guidelines of the International Federation for the Surgery of Obesity (IFSO), the International Federation for the Surgery of Obesity—European Chapter (IFSO-EC), the European Association for the Study of Obesity (EASO) [23] and the Bariatric Chapter of the Association of Polish Surgeons [24].

The following associated medical problems were observed preoperatively in our cohort: hypertension (HA) (*n* = 42; 45.2%), hypercholesterolemia (*n* = 24; 25.8%), active smoking (*n* = 23; 24.7%) and type 2 diabetes mellitus (t2dm) (*n* = 11; 11.8%).

### 2.2. Inclusion Criteria

All patients aged 18 years or older who were qualified for SG based on clinical and surgical eligibility criteria and who provided informed consent were included in the study. Eligibility criteria included a BMI meeting surgical thresholds for obesity management, absence of contraindications for laparoscopic surgery and the ability to comply with postoperative follow-up requirements.

### 2.3. Exclusion Criteria

Patients were excluded if they had undergone previous bariatric or gastric surgery, lacked information about prior Helicobacter pylori infection status, had incomplete follow-up data or presented with severe comorbidities (such as advanced cardiovascular disease, active malignancies, or other conditions that could significantly impact surgical outcomes or long-term follow-up).

### 2.4. Data Gathering

This study involved retrospective and prospective data collection from eligible patients. The extracted information comprised demographic data; surgical outcomes and follow-up results, including complications, body weight and BMI 3, 6 and 12 months after SG.

### 2.5. Definition of Complications

Any deviations from the normal postoperative course, whether requiring treatment or not, were categorized into groups in this study. These groups included mineral and vitamin deficiencies, incidence of hypoglycemia, gastrointestinal motility disorders and any other abnormalities observed in laboratory tests (for example, elevated bilirubin, etc.) If there were any cases of surgical complications, they were reported using the Clavien–Dindo scale [25].

### 2.6. HP Eradication

All patients underwent gastroscopy prior to the operation, during which a mucosal sample from the stomach was taken for the rapid urease test (H.p. Swift Test, Instytut Żywności i Żywienia, Poland) to screen for HP; the result was obtained after 30 min. For patients who tested positive, eradication was recommended. Additionally, if the HP test on the postoperative specimen yielded positive results, patients underwent treatment. The administered treatment regimens align with the recommendations outlined in the Maastricht IV/Florence Consensus Report [18] and are presented in Table 1.

### 2.7. Surgical Technique

A laparoscopic approach was used to reach the abdominal cavity. The dissection of short gastric vessels was conducted, starting from about 6 cm anterior to the pyloric valve and continuing to the point of the angle of His. A stapler technique based on a 34 Fr calibrating tube was used to create a gastric pouch [26].

### 2.8. Pathology Examination

The dissected part of the stomach was fixed in 10% buffered formalin and transferred to the Department of Pathomorphology. After processing, the specimens underwent hematoxylin–eosin staining, modified Giemsa staining and immunohistochemical staining for the presence of HP. The microscopic examination was performed with full access to patient medical history.

### 2.9. Statistical Analysis

All statistical calculations were performed using the StatSoft Inc. (2014) statistical package STATISTICA (version 12.0. Armonk, NY, USA).

Quantitative variables were characterized by the arithmetic mean, standard deviation, median, minimum and maximum (range) and 95% CI (confidence interval). Qualitative variables, on the other hand, were presented with the use of frequencies and percentages. The Shapiro–Wilk W test was used to check whether each quantitative variable came from a normally distributed population. Levene’s test or the Brown–Forsythe test was used to test the hypothesis of equal variances. The significance of differences between the two groups (model of unrelated variables) was tested by use of Student’s *t*-test (or, in the absence of homogeneity of variance, Welch’s *t*-test) or the Mann-Whitney U test (in the case of failure to meet the applicability conditions of Student’s *t*-test or for variables measured on an ordinal scale). The significance of differences among more than two groups was checked by the F test (ANOVA) or Kruskal–Wallis test (if the conditions of ANOVA applicability were not met). In the case that statistically significant differences were found between the groups, post hoc tests were used (Tukey’s test for the F test, Dunn’s test for Kruskal–Wallis). In the case of a model of two related variables, Student’s *t*-test or the pairwise Wilcoxon test was used (in the case of failure to meet the conditions of applicability of Student’s t-test or for variables measured on an ordinal scale). The significance of differences among more than two related variables in a model of related variables was checked by repeated-measures analysis of variance or Friedman’s test (if the conditions of applicability of repeated-measures analysis of variance or for variables measured on an ordinal scale were not met). Chi-square tests of independence were used for categorical variables (using the Yates correction for cell counts below 10, checking Cochran’s conditions and Fisher’s exact test, respectively). To establish the strength and direction of relationships between the variables, correlation analysis was applied by calculating the Pearson and/or Spearman correlation coefficients. In all calculations, the significance level was *p* = 0.05.

## 3. Results

Between May 2020 and May 2021, 265 patients underwent operations at the bariatric center, among which 144 were SG procedures. A total of 51 patients met our exclusion criteria (13 lost to follow-up, 38 follow-ups incomplete); therefore, these patients were not included in our study. The remaining 93 patients (69 female; 74.2%) were enrolled in the study. Based on the presence of HP infection in stomach samples, the study group was divided into two subgroups: A (n = 11; presence of HP infection) and B (n = 82; lack of HP infection). The baseline characteristics of the study groups are presented in Table 2.

The mean body weight and BMI in our cohort at the time of operation were 113.1 kg (SD = 19.7) and 38.9 kg/m^2^ (SD = 5.3), respectively. There were no significant differences between study groups in terms of operational body weight (*p* = 0.2116) and BMI (*p* = 0.5323). In group A, which included individuals who tested positive for HP, there was a significant decrease in body weight at 6 and 12 months following surgery compared to the initial body weight (*p* < 0.0001), but not at 3 months. By contrast, in group B, there was a significant decrease in body weight at 3, 6 and 12 months following surgery compared to the initial body weight (*p* < 0.0001). In group A, there was a significant decrease in BMI at 6 months and 12 months following surgery compared to the baseline value (*p* = 0.0001), but not at 3 months. In group B, there was a significant decrease in BMI at 3, 6 and 12 months following surgery compared to the baseline value (*p* < 0.0001). Additionally, in group B, BMI further decreased significantly at 6 and 12 months compared to BMI at 3 months after surgery.

The analysis of the data did not demonstrate any statistically significant differences in body weight at 3 months (*p* = 0.3757), 6 months (*p* = 0.1422) or 12 months (*p* = 0.2737) after surgery between the study groups. Similarly, there were no statistically significant differences in BMI at 3 months (*p* = 0.7026), 6 months (*p* = 0.3957) or 12 months (*p* = 0.8265) post-surgery in relation to HP infection.

The overall incidence rates of side effects related to surgery are presented in Table 3. There were no statistically significant differences between the groups. The occurrence of side effects was documented during the fourth follow-up visit after the operation.

In group B, we observed one surgical complication, marked as 3b in the Clavien–Dindo classification [25], that required reoperation because of recurrent pain in the upper abdomen. In contrast, no complications were observed in group A.

Table 4 shows the incidence of side effects after a 12-month postoperative period. Among other disturbances in laboratory parameters, we included changes in the hepatobiliary profile and endocrine disorders. The latter are due to an insufficient endocrine care system for patients after bariatric surgery and inappropriate dosages based on patients’ body weight.

We observed no statistically substantial differences in body weight loss between groups after a 12-month postoperative follow-up period, which is in line with previous publications [27,28].

**Figure 1:**
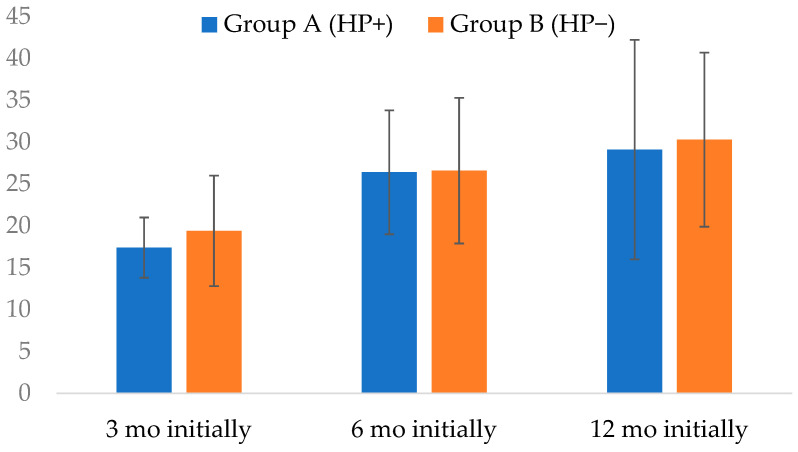
Comparative characterization of the studied groups in terms of body weight loss at 3, 6 and 12 months post-surgery compared to the baseline values.

As indicated in Figure 1, there was a significant decrease in body weight in both groups at 6 and 12 months following surgery. However, in the group with HP infection, the loss was not as statistically significant 3 months after SG as in the HP negative group. The body weight reduction may have been disturbed due to infection. The pathophysiology of this phenomenon might be multifaceted. An active infection in a patient may have an impact on the homeostasis of hormones and mediators. Among these are leptin and ghrelin, which affect food intake and energy balance [29]. An active infection could lead to the incidence of early postoperative foregut symptoms [30], which may influence daily dietary habits. Furthermore, the inflammation accompanying the infection changes gastric motor function, which enhances gastric compliance [3]. Antibiotic eradication was recommended in this period. Therefore, it is important to note that the use of antibiotics can also have potential side effects, including nausea and vomiting [31].

## 4. Discussion

In our study, HP infection appeared to have no significant impact on the overall ratio of postoperative side effects, including surgical complications. However, the infected group did exhibit a higher percentage of patients with gastrointestinal motility disorders than the uninfected group. This included postoperative gastroesophageal reflux disease (GERD), which was found to occur more frequently in the HP+ group [32]. Due to the unclear results, further studies with a larger population are necessary to determine the precise relationship between HP infection and gastrointestinal motility disorders.

In this study, the prevalence of HP infection was 11.83%, based on postoperative specimens. This percentage refers to patients who probably had ineffective HP eradication due to various factors [33], one of which is the possibility of re-infection in view of the moderate to high incidence of HP infection in the Polish population [34]. Additionally, there are still treatment variants that include clarithromycin, despite the rising bacterial resistance to this antibiotic [35]. Moreover, the rapid urease test has been proven to have lower sensitivity than histopathologic examination in diagnosing HP infection [36]. Unfortunately, many patients still do not undergo evaluation for effective HP eradication through stool antigen detection or a 13-C urea breath test, which are included in the recommended guidelines [16].

Although the question arises of whether there is a need for screening and HP eradication prior to surgery, on account of various factors and hazards, these procedures are crucial and cannot be excluded. One of the most common complications after SG is gastroesophageal reflux. There is evidence that HP has an influence on the increased incidence of postoperative GERD [32]. Thus, it contributes to prolonged therapy using PPI in patients, which could lead to atrophic gastritis. This condition is proven to eventually progress to gastric cancer [37].

Interestingly, despite HP’s recognized effects on gastric physiology, including its influence on leptin and ghrelin levels, as well as gastric motility, we did not observe significant differences in body weight or BMI changes between the HP+ and HP− groups at 3, 6 or 12 months postoperatively. This highlights the complexity of HP’s role in metabolic and bariatric outcomes, possibly mediated by individual patient factors or the timing of infection relative to surgical intervention.

Additionally, HP infection might indirectly affect surgical outcomes through its influence on inflammation and nutrient absorption. For example, chronic inflammation caused by HP could impair gastric healing or exacerbate deficiencies of vitamins and minerals essential for recovery [38]. Although not prominent in our study, the higher rates of deficiencies in the HP+ group warrant closer investigation.

Future research should focus on refining diagnostic techniques and tailoring eradication protocols to address regional antibiotic resistance patterns. Studies with extended follow-up and larger sample sizes are crucial to elucidate the long-term effects of HP infection on bariatric outcomes. Furthermore, exploring the interplay between HP and factors such as microbiome alterations, hormonal regulation and immune responses post-SG could provide deeper insights.

## 5. Conclusions

Active HP infection does not appear to significantly influence the overall rates of postoperative complications or weight loss outcomes in the 12-month period following SG. However, its presence may contribute to an increased incidence of gastrointestinal motility disorders including GERD, highlighting a potential area of concern for long-term postoperative management.

While HP infection did not result in statistically significant differences in BMI reduction or weight loss between the infected and non-infected groups, the slower rate of weight reduction observed in the initial three months among HP+ patients suggests that the infection might influence early recovery phases.

Further research is necessary to clarify the mechanisms underlying HP’s impact on gastric physiology and bariatric outcomes. Larger cohort studies with extended follow-up periods should focus on exploring the interplay among HP infection, postoperative hormonal changes and gastrointestinal motility to refine perioperative management strategies.

## Figures and Tables

**Table 1:** Scheme of HP infection therapy.

Therapy Type	Medications	Duration
First-line therapy A. Triple therapy without clarithromycin	PPI—standard dose 2 times a day	
	Amoxicillin—2 times 1.0 g	10 days
	Metronidazole—2 times 0.5 g	
B. Quadruple therapy with bismuth	PPI—standard dose 2 times a day	
	Bismuth citrate—2–4 times 0.120 g Tetracycline—4 times 0.5 g Metronidazole—3 times 0.5 g	10–14 days
C. Sequential therapy (not recommended)	Days 1–5:	
	PPI—standard dose 2 times a day	10 days
	Amoxicillin—2 times 1.0 g	
	Days 6–10: PPI—standard dose 2 times a day Clarithromycin—2 times 1.0 g Tinidazole/metronidazole—2 times 0.5 g	
	PPI—standard dose 2 times a day Amoxicillin—2 times 1.0 g Clarithromycin—2 times 0.5 g Tinidazole/metronidazole—2 times 0.5 g	14 days
Second-line therapy A. Quadruple therapy with bismuth	(Same as above)	(Same as above)
B. Sequential therapy	(Same as above)	(Same as above)
C. Triple therapy with levofloxacin	PPI—standard dose 2 times a day Amoxicillin—2 times 1.0 g Levofloxacin—2 times 0.25 g	10–14 days

PPI—proton pump inhibitors.

**Table 2:** Basic characteristics of the studied groups in terms of sex, age, body weight and BMI at baseline.

	Group A (HP+) (*n* = 11)	Group B (HP−) (*n* = 82)	All (*n* = 93)	*p*-Value
**Sex**				0.5383 ^1^
female	9 (81.8%)	60 (73.2%)	69 (74.2%)	
male	2 (18.2%)	22 (26.8%)	24 (25.8%)	
Age [years]				0.7259 ^1^
mean (SD)	41.2 (8.7)	42.2 (9.1)	42.1 (9.0)	
range	26.0–57.0	26.0–65.0	26.0–65.0	
median	42.0	42.0	42.0	
95% CI	[35.4; 47.0]	[40.2; 44.2]	[40.2; 43.9]	
Body weight [kg]				0.2116 ^2^
mean (SD)	110.5 (26.6)	113.5 (18.8)	113.1 (19.7)	
range	85.0–168.0	86.0–195.0	85.0–195.0	
median	100.0	110.0	109.0	
95% CI	[92.6; 128.3]	[109.3; 117.6]	[109.0; 117.0]	
BMI [kg/m^2^]				0.5323 ^2^
mean (SD)	38.4 (6.9)	39.0 (5.1)	38.9 (5.3)	
range	29.4–50.3	30.4–58.9	29.4–58.9	
median	35.9	38.1	38.1	
95% CI	[33.7; 43.0]	[37.9; 40.1]	[37.8; 40.0]	

^1^ Chi-square, ^2^ Mann–Whitney U; BMI—body mass index.

**Table 3:** Comparative characteristics of the studied groups in terms of the incidence of side effects.

	Group A (HP+) (*n* = 11)	Group B (HP–) (*n* = 82)	All (*n* = 93)	*p*-Value
3 mo.	2 (18.2%)	26 (31.7%)	28 (30.1%)	0.3585 ^1^
6 mo.	6 (54.5%)	33 (40.2%)	39 (41.9%)	0.3667 ^1^
12 mo.	7 (63.6%)	35 (42.7%)	42 (45.2%)	0.1898 ^1^

^1^ Chi-square.

**Table 4:** Incidence of side effects in both groups after a 12-month postoperative period.

	Group A (HP+) (*n* = 11)	Group B (HP−) (*n* = 82)	All (*n* = 93)
Hypoglycemia	0 (0%)	4 (4.88%)	4 (4.30%)
Gastrointestinal motility disorders	1 (9.09%)	11 (13.41%)	12 (12.90%)
Mineral and vitamin deficiencies	3 (27.27%)	4 (4.88%)	7 (7.52%)
Anemia	0 (0%)	2 (2.44%)	2 (2.15%)
Other disturbances in laboratory parameters	0 (0%)	6 (7.32%)	6 (6.45%)
Other side effects, unclassified	4 (36.36%)	19 (23.17%)	23 (24.73%)

## Data Availability

The data that support the findings of this study are available from the corresponding author, M.S., upon reasonable request.
